# Specific Deletions of Chromosomes 3p, 5q, 13q, and 21q among Patients with G2 Grade of Non-Small Cell Lung Cancer

**DOI:** 10.3390/ijms25168642

**Published:** 2024-08-08

**Authors:** Agata Kolecka-Bednarczyk, Magdalena Frydrychowicz, Bartłomiej Budny, Marcin Ruciński, Claudia Dompe, Piotr Gabryel, Bartosz J. Płachno, Marek Ruchała, Katarzyna Ziemnicka, Paweł Zieliński, Joanna Budna-Tukan

**Affiliations:** 1 Department of Immunology, Poznan University of Medical Sciences, 60-806 Poznan, Poland; frydrychowicz@ump.edu.pl (M.F.); 84416@student.ump.edu.pl (C.D.); 2 Department of Endocrinology, Metabolism and Internal Medicine, Poznan University of Medical Sciences, 60-355 Poznan, Poland; bbudny@ump.edu.pl (B.B.); mruchala@ump.edu.pl (M.R.); kaziem@ump.edu.pl (K.Z.); 3 Department of Histology and Embryology, Poznan University of Medical Sciences, 60-781 Poznan, Poland; marcinruc@ump.edu.pl (M.R.); jbudna@ump.edu.pl (J.B.-T.); 4 Doctoral School, Poznan University of Medical Sciences, 60-812 Poznan, Poland; 5 Department of Thoracic Surgery, Poznan University of Medical Sciences, 60-569 Poznan, Poland; pgabryel@ump.edu.pl (P.G.); pzielinski986@gmail.com (P.Z.); 6 Department of Plant Cytology and Embryology, Institute of Botany, Faculty of Biology, Jagiellonian University in Kraków, 30-387 Cracow, Poland; 7 Department of Anatomy and Histology, Collegium Medicum, University of Zielona Gora, 65-046 Zielona Gora, Poland

**Keywords:** lung cancer, microarrays, loss of heterozygosity, gene enrichment

## Abstract

Non-small cell lung cancer (NSCLC) leads as a primary cause of cancer-related premature mortality in Western populations. This study leverages cutting-edge gene-expression-profiling technologies to perform an in-depth molecular characterization of NSCLC specimens, with the objective of uncovering tumor-specific genomic alterations. By employing DNA microarray analysis, our research aims to refine the classification of NSCLC for early detection, guide molecular-targeted treatment approaches, enhance prognostication, and broaden the scientific understanding of the disease’s biology. We identified widespread genomic abnormalities in our samples, including the recurrent loss of chromosomal regions 3p, 5q, 13q, and 21q and the gain of 12p. Furthermore, utilizing Metascape for bioinformatic analysis revealed critical biological pathways disrupted in NSCLC, offering promising leads for novel therapeutic interventions.

## 1. Introduction

Lung cancer is the most common neoplasm in both men and women. In 2018, lung cancer was globally the most diagnosed cancer, with 2,094,000 new cases. It ranks second in both men and women, with 1,369,000 and 725,000 cases, respectively. Developing nations with high smoking rates show the highest incidence, with lung cancer being the most common cancer in 37 nations [1]. In 2019, lung cancer was the seventeenth leading cause of disability-adjusted life years (DALYs) overall. However, it ranked much higher as the fifth leading cause for the age group 50 to 74 and the seventh leading cause for those aged 75 years and older in terms of DALYs [2]. The variable risk of developing lung cancer depends primarily on active or passive exposure to the carcinogenic components of tobacco smoke (about 90% of all cases) [3]. Despite many clinical trials, modern diagnostic techniques, and improved supportive care, most patients are diagnosed late, at an advanced stage. Additionally, long-term survival has not increased over the last decades [4].

Based on histological verification and tumor biology, lung cancer is classified into two major groups: small cell lung carcinoma (SCLC), accounting for about 20% of all primary lung cancers, and non-small cell lung carcinoma (NSCLC), accounting for about 75%. NSCLC is further divided into squamous cell carcinoma (SCC) and non-squamous cell carcinoma (non-SCC) with distinctive subtypes: adenocarcinoma (AC) and large cell carcinoma (LCC) [5]. Lung cancer, particularly NSCLC, is a genetically complex disease, developing because of the accumulation of multiple genetic abnormalities. This group of lung tumors is biologically heterogeneous, and genomic dysregulation affects cell division and replication. Numerous genetic/epigenetic factors, especially genetic polymorphisms, copy number alteration, or epigenetic modifications, have been extensively investigated in lung cancer [6,7]. The DNA sequence of NSCLC cells undergoes alterations from an early progenitor cell, granting them a growth advantage. These changes include small point mutations and large genomic rearrangements, including variations in ploidy. These molecular alterations directly impact the aggressiveness of cancer phenotypes and, subsequently, affect patient outcomes. For example, loss of heterozygosity (LOH) is a major mechanism inactivating tumor suppressor genes, and it is involved in the process of neoplasm formation [8].

Molecular studies like genomic microarrays and allelic imbalance analysis offer novel approaches for assessing lung tumors beyond conventional pathology, providing insights into NSCLC’s prognostic relevance, identifying early diagnostic biomarkers, and unveiling therapeutic targets. The gene ontology (GO) enrichment analysis conducted in this study further enhances our understanding of the functional implications of genomic abnormalities in NSCLC samples by associating them with specific biological processes and pathways related to lung cancer, potentially revealing new therapeutic targets. This study aims to comprehensively characterize three representative NSCLC samples using DNA microarray analysis, refining classification for early detection, guiding molecular treatments, addressing prognostic concerns, and deepening our understanding of lung cancer biology.

## 2. Results

The strategy for a reliable detection of chromosomal rearrangements assumes the usage of the CytoScan 750K chip. This investigation resulted in the detection of ~62 genomic imbalances that were not filtered out and passed the genomic criteria (>400 Kb; 50 markers). The genomic regions were then divided into four groups: low mosaic gains (CN2-3), low mosaic losses (CN2-1), amplifications (CN > 3), and deletions suggestive for the loss of heterozygosity (CN < 1).

The common lesions in all three patients encompassed deletions in 3p, 5q, 13q, and 21q and the amplification in 12p chromosomes. LOH lesions in all three patients were found on chromosome 3, 7, and X. The identification of the clinically relevant region was based on genomic coordinates.

The loci presenting deletions (3p, 5q, 13q, and 21q) were further elucidated for cancer relevance. The analysis focused on census genes located within certain regions. In total, 59 census genes were established. All genes were then analyzed for their functional interactions and potential pathways contributing to disease. The analysis identified three significant clusters of regulatory factors, namely, RB1, BRCA2/APC, and PBRM1, involved in chromatin remodeling, highlighting the pivotal role of negative regulators of cell proliferation.

**Figure 1:**
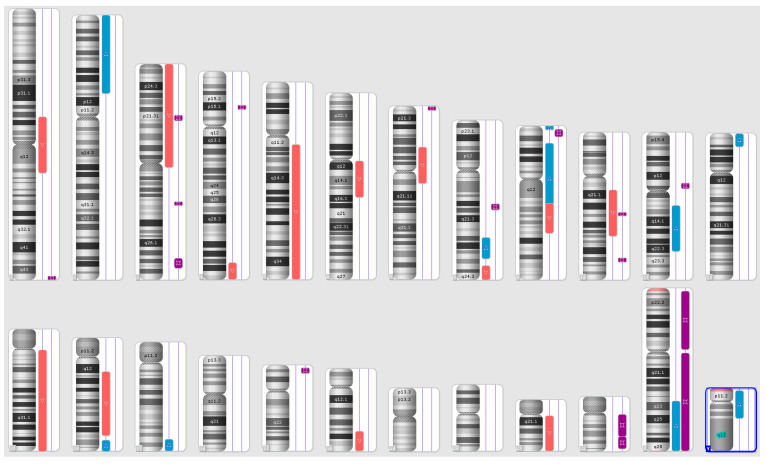
A karyoview depicting the identified genomic abnormalities (on the right side of each ideogram) in the cancer tissue of patient 1. Gains are represented by blue bars, losses are similarly depicted in red bars, and regions of loss of heterozygosity (LOH) are denoted by violet bars.

**Figure 2:**
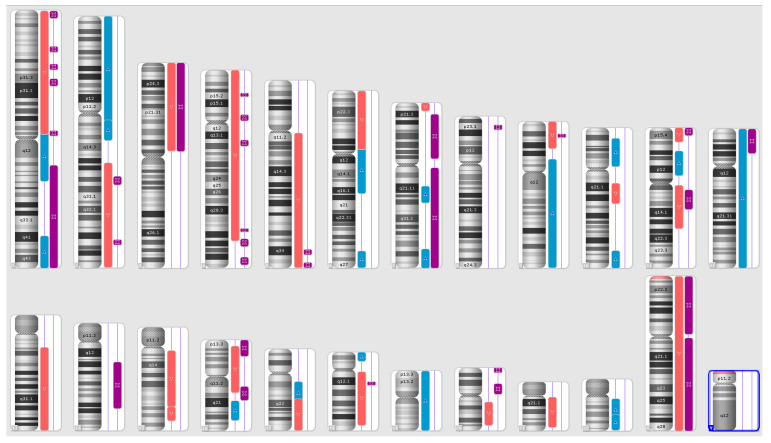
A karyoview depicting the identified genomic abnormalities (on the right side of each ideogram) in the cancer tissue of patient 2. Gains are represented by blue bars, losses are similarly depicted in red bars, and regions of loss of heterozygosity (LOH) are denoted by violet bars.

**Figure 3:**
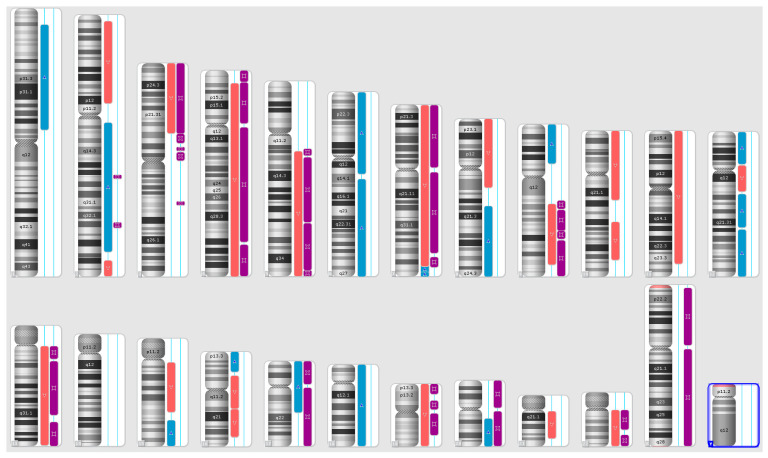
A karyoview depicting the identified genomic abnormalities (on the right side of each ideogram) in the cancer tissue of patient 3. Gains are represented by blue bars, losses are similarly depicted in red bars, and regions of loss of heterozygosity (LOH) are denoted by violet bars.

**Figure 4:**
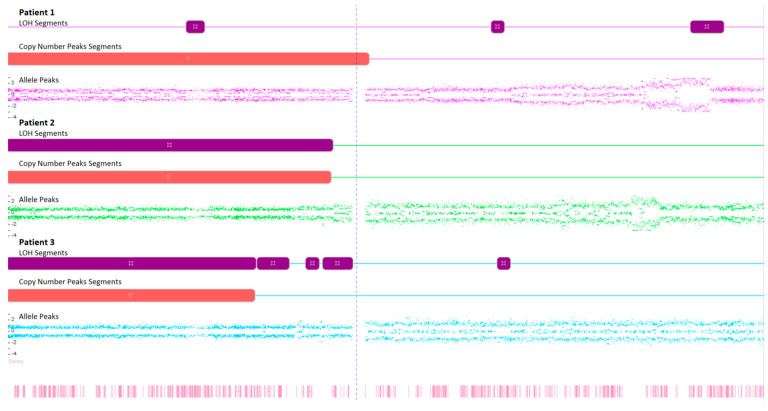
Microarray analysis of chromosome 3 in examined FFPA samples, illustrating the loss of heterozygosity (LOH), copy number state, and Log2 ratio.

Karyograms illustrating the localization of each segment are shown in Figure 1, Figure 2 and Figure 3, and an example of mosaic aneuploidy (chromosome 3) is shown in Figure 4. All of these data and precise genomic coordinates were incorporated into Table 1, Table 2 and Table 3, respectively.

To determine the potential function of the relevant genes from Cancer Gene Census, we conducted functional analysis using the Metascape bioinformatic tool v3.5. Metascape offers a comprehensive suite of analysis tools, including functional enrichment analysis, interactome analysis, gene annotation, transcriptional factor–gene interaction, and membership search with more than 40 independent knowledge databases.

**Figure 5:**
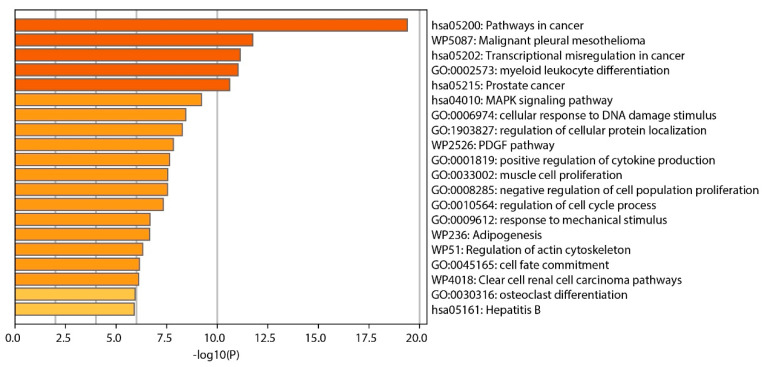
Bar graph displaying enriched terms derived from input gene lists, with bars color-coded according to their *p*-value significance.

Our analysis identified several clusters of enrichment terms, with the most significant being “Pathways in cancer” (category: KEGG Pathway, log10(*p*) = −19.4), “Malignant pleural mesothelioma” (category: WikiPathways, log10(*p*) = −7.7), and “Transcriptional misregulation in cancer” (category: KEGG Pathway, log10(*p*) = −7.27), as shown in Figure 5.

**Figure 6:**
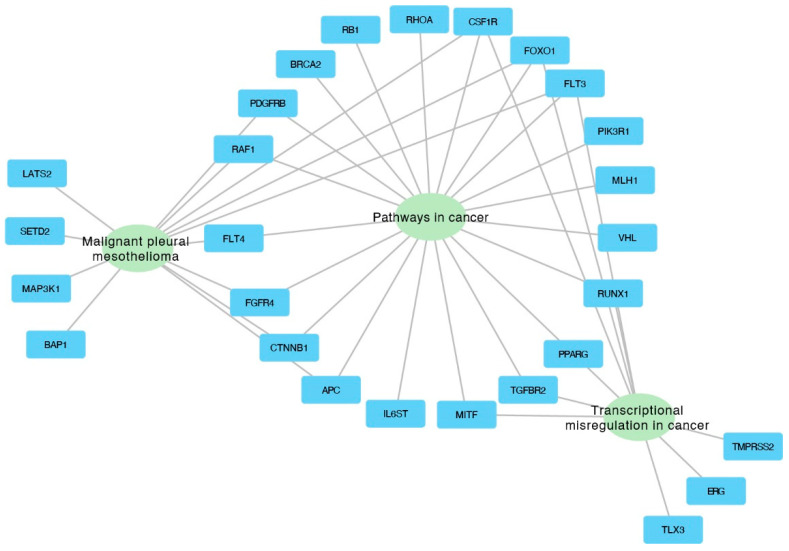
Interaction network depicting the top three enriched categories and the associated genes within each category.

The genes from the three most significantly enriched categories were visualized using Cytoscape, which illustrates the assignment of genes to specific categories through connecting edges (Figure 6).

**Figure 7:**
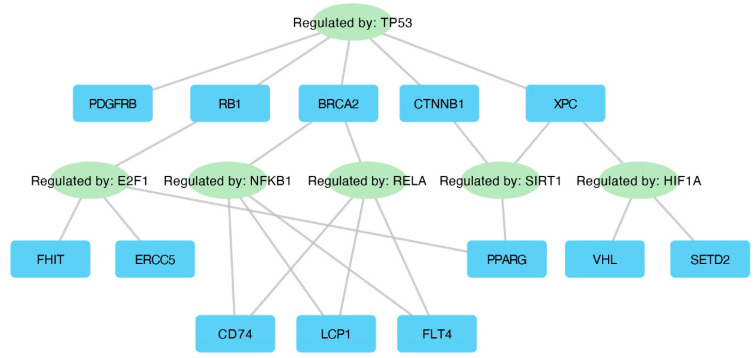
Interaction network displaying key transcription factors and their corresponding target genes.

The TRRUST database (Transcriptional Regulatory Relationships Unravelled by Sentence-based Text mining) was utilized to explore interactions between transcription factors and their target genes. The results of this analysis are presented in Figure 7.

## 3. Discussion

Lung cancer is characterized by multiple quantitative changes, such as aneuploidy, and structural changes, such as amplifications and deletions at the chromosomal level. The genetic analysis of the two most common types of NSCLC, adenocarcinoma and squamous cell carcinoma, showed several point mutations, including “driving mutations” initiating proliferation cascades. As a part of the project, The Cancer Genome Atlas (TCGA) identified lung cancer’s most frequent genetic alterations, including mutation, rearrangement, or amplification. In adenocarcinoma, the major contributors encompass EGFR, KRAS, TP53, LRP1B, CDKN2A, EML4-ALK, MET, ROS1, RET, and BRAF, while in squamous cell carcinoma, they encompass TP53, EGFR, LRP1B, NFE2L2, CDKN2A, FAT4, PIK3CA, KMT2C, KRAS, and PTEN [9]. The impact of genetic alterations in NSCLC centers around genes encoding receptor tyrosine kinases (RTKs), whose activation leads to a heightened proliferation, survival, migration, and invasion of cancer cells. These alterations are frequent in EGFR, ALK, MET, ROS1, RET, FGFR1, and DDR2 genes, as well as in the genes coding proteins facilitating the transmission of RKT, such as KRAS, BRAF, and PIK3CA. Point mutations and deletions in PTEN occur in 15–20% of squamous cell carcinomas, resulting in an abnormal activation of the PI3K pathway. However, genetic changes in RKT proteins are different in adenocarcinoma and squamous cell carcinoma. EGFR and KRAS are two of the most mutated genes in adenocarcinomas, while these genes rarely change in squamous cell carcinoma [10,11,12].

In this study, we documented numerous copy number variation (CNV) lesions among three representative patients, with each displaying a unique pattern of chromosomal arm amplifications and deletions. The most prevalent genetic alterations shared among the patients were losses in the 3p, 5q, 13q, and 21q regions, along with gains in the 12p region. Notably, the 3p chromosome change impacts over 500 genes, featuring tumor suppressors like BAP1, FHIT, VHL, PTPγ, SEMA3B, SEMA3F, TUSC2, and MLH1. Chromosome 5 presented alterations in approximately 800 genes, including the suppressor genes APC, MCC, FER, and IRF1, as well as transcription factors like alpha and beta interferons, which are also involved in apoptotic regulation [13,14].

The deletion on chromosome 13 affects about 400 genes, including BRCA2. On chromosome 9, the amplification of the CDKN2A suppressor gene was identified in one patient, while a deletion was identified in another. CDKN2A regulates two critical cell cycle pathways associated with p53 and RB1 proteins [15,16]. In two patients, we observed a deletion in the EGFR gene region on chromosome 7. EGFR, part of the ErbB family of receptors, exhibits tyrosine kinase activity and is overexpressed on the surface of tumor cells in various cancers, including lung cancer [17]. In two patients, we identified an amplification on chromosome 12, including the KRAS gene. Furthermore, we observed changes in genes associated with antigen presentation and the immune system response, including NOTCH2, CUL3, FOXP1, NFE2L2, HLA-A, IL-2, CD83, TNF, HLA-DRA, CD209, ICAM1, TGFB1, and CD40 [18].

Numerous studies by other authors confirmed the utility of CNV analysis in clinical settings. Heo at al. compared CNV changes in the peripheral blood of patients with NSCLC with healthy controls. Five nominally significant associations were found between the NSCLC and CNVs. As the result of the study, 979 CNVs were acquired, with 582 copy-number gains and 967 copy-number losses, showing that CNV analysis is promising in the search for lung cancer biomarkers [19]. Huang et al. found that the number of CNV changes in NSCLC increases with disease progression. The genes in the regions with alterations are functionally related to each other or belong to the same signaling pathways [20]. In their study, Li et al. compared the number of CNV changes between primary lung adenocarcinoma and secondary brain metastasis using comparative genomic hybridization. CNV changes were significantly higher in the brain metastasis than in the primary tumor [21]. An analysis of CNV changes can contribute to a better understanding of cancer mechanisms. CNV can be employed in monitoring disease progression, and it may influence early lung cancer diagnosis. A study showed that circulating tumor cells (CTCs) derived from the peripheral blood of patients with lung cancer showed characteristic CNV lesions like those occurring in metastatic lesions. Different patients with adenocarcinoma had the same CNV changes, while patients with SCLC had different CNV changes. The authors suggested that some CNV changes are predictors of metastatic changes, and their detection in CTC can be a useful diagnostic tool [22]. In his work, Bowcock also suggested the diagnostic utility of CNV changes on chromosome 3, specifically at loci 3p26-p11.1 (deletion), 3q26.2-29 (amplification), and 6q25.3-24.3 (deletion), previously associated with endobronchial squamous metaplasia. These CNV changes demonstrated 97% specificity in patients diagnosed with lung cancer within 44 months. This study detected CNV changes in nine cases, accurately predicting the phenotype in 92% of cases. These results confirmed the diagnostic importance of these chromosome 3 regions, particularly the amplification of locus 3q26.2-q29 harboring the PIK3CA gene, a vital cell growth regulator [23]. In terms of treatment, He at al. proposed the use of CNV as a prognostic marker for patients with advanced NSCLC. It was noted that 1p and 14q gains are associated with opposite TKI efficacy in EGFR-positive patients and the gain in the 7q region with prolonged PFS and OS in a group of EGFR-negative patients. These results may help to predict the response to EGFR-TKI treatment and additionally assess the risk of relapse, improving treatment efficacy in NSCLC patients [24]. Further investigations with larger cohorts of NSCLC patients are warranted to validate these findings and explore CNV alterations in other genome regions. 

The loss of heterozygosity (LOH) is the primary mechanism inactivating tumor suppressor genes and is involved in neoplasm formation. Some of the most identified LOH alterations in lung cancer are located on chromosomes 1p, 2q, 3p, 8p, 9p, 17p (the locus of the Tp53 suppressor gene), 18q, and 19p. The LOH of chromosomes 3p14, 9p21, and 17p13 are often observed in the bronchial epithelium of smokers, and their incidence is mainly linked to in situ cancer. The deletion of the 3p chromosome is also observed at an early stage in the pathogenesis of many other cancers, including head and neck, cervix, and breast cancers [25,26,27]. We identified LOH changes showing high genomic instability and localization variability in each examined patient. These alterations predominantly affected chromosomes 3, 7, and X. Notably, a LOH in the 3p region is the most frequent and earliest genetic change implicated in the pathogenesis of lung cancer, with significant implications for disease development, as supported by extensive research. LOH changes in the 3p locus are associated with a worse prognosis for patients with adenocarcinoma. Different studies confirm a connection between the occurrence of LOH on 3p and the shorter overall survival of patients with NSCLC [28,29].

The functional gene enrichment analysis conducted in the study identified several significantly enriched gene ontology (GO) terms, illuminating the functional consequences of the genomic abnormalities discovered in NSCLC samples. This analysis links specific genetic abnormalities to biological processes and pathways crucial to lung cancer, enhancing our understanding of the disease’s molecular mechanisms. From an analysis of 719 oncogenes in the cancer gene census, 59 genes were identified as commonly absent in the 3p, 5q, 13q, and 21q regions. The gene enrichment analysis revealed several significantly enriched GO terms, with three closely associated with lung cancer, including “Pathways in cancer”, “Malignant pleural mesothelioma”, and “Transcriptional misregulation in cancer”. These GO terms encompass six genes relevant to lung cancer found primarily in “Pathways in cancer” GO and partially overlapping with two other GOs: FLT3, FOXO1, CSF1R, RUNX1, PPARG, and TGFBR2.

FMS-related receptor tyrosine kinase 3 (FLT3), a class III receptor tyrosine kinase family member, is primarily expressed in early myeloid and lymphoid progenitors and plays a vital role in their proliferation and differentiation [30]. FLT3 promotes the activation of downstream pathways involving PI3K, AKT, mTOR, RAS, and extracellular signal-related kinase [31]. FLT3 mutations were primarily studied in acute myeloid leukemia [32]. FLT3 internal tandem duplication (FLT3-ITD) is among the two most common driver mutations associated with poor prognosis [33]. Different studies identified mutations in NSCLC and SCLC patients [34,35]. FLT3 is a target for other therapies, and inhibitors have already been developed for precise treatments and overcoming acquired resistance [36,37]. Ryu et al. showed that FLT3 inhibitors, combined with other therapies, displayed a synergistic effect in suppressing NSCLC proliferation [38]. Abrams et al. reported that an oral multitargeted tyrosine kinase inhibitor directly targeting FLT3 might aid in treating SCLC [39].

Colony stimulating factor 1 (CSF1) signaling through its receptor, CSF1R, plays a pivotal role in the proliferation and differentiation of myeloid-derived suppressor cells (MDSCs) and M2-type tumor-associating macrophages (M2-TAMs) [40]. MDSCs contribute to immune evasion and tumor progression by activating M2-TAMs and regulatory T cells [41,42]. Despite the complexity of the CSF1R pathway, recent transcriptomic analyses have identified potential targets to modify TAM recruitment and polarization [43]. Targeting CSF1 signaling through inhibitors presents a therapeutic strategy to disrupt MDSC proliferation and survival [44]. Combining CSF1R inhibitors with fibroblast growth factor receptors (FGFRs) offers a promising approach to reducing MDSC infiltration and tumor progression [45]. Clinical trials targeting CSF1/CSF1R in solid tumors have shown modest efficacy despite their ability to deplete macrophages [46]. Overall, conflicting evidence exists regarding the role of CSF1R-expressing TAMs in lung cancer, with some studies suggesting their prognostic neutrality or limited impact on tumor growth [47]. These findings underscore the need for alternative strategies to target macrophages and improve therapy in NSCLC, as CSF1R inhibition may not be the optimal approach [43]. CSF1R was found to be differently mutated in many lung cancer patients affected explicitly by NSCLC and SCLC [47]. A study found that the CSF1 modulation of TAM and tumor progression is affected by the transcription factor forkhead box protein O1 (FOXO1). This transcription factor mediates the polarization of M0 macrophages towards the M2 phenotype, a process pivotal in tumor progression and immune evasion [48].

FOXO1 regulates various cellular metabolic processes [49]. While FOXO1 traditionally acts as a tumor suppressor, its regulation and function in lung cancer are complex, influenced by intricate networks of signaling pathways, post-transcriptional modifications, protein stability, and interactions with upstream regulators [50]. Studies have shown that FOXO1 activity is modulated by phosphorylation, methylation, acetylation, and ubiquitination, affecting its tumor-suppressive or oncogenic functions in lung carcinogenesis [51]. Various signal transduction pathways can regulate FOXO1 activity under stress conditions, and subsequent post-transcriptional modifications can induce or inhibit FOXO1 activity [52]. Additionally, FOXO1 interacts with pivotal pathways like the PI3K/AKT axis, where dysregulation contributes to tumor progression and therapeutic resistance [53,54]. Understanding the interplay between FOXO1 and upstream regulators like DNA damage response mechanisms, epithelial-to-mesenchymal transition, and non-coding RNAs provides valuable insights into potential therapeutic strategies. FOXO1 has emerged as a new therapeutic target in NSCLC, as its enhanced transcriptional activity is associated with increased resistance to gefitinib [55]. Conversely, the knockdown of FOXO1 reduces cellular sensitivity to cisplatin, another chemotherapy drug used in NSCLC treatment [56].

Mutations of the runt-related transcription factor 1 (RUNX1), a member of the RUNX family proteins essential for hematopoiesis, have been implicated in various solid tumors, including lung cancer [57,58]. Analyses of methylation status and expression levels of RUNX1 in NSCLC patients reveal its potential clinicopathological significance, offering insights into its role as a diagnostic biomarker and a therapeutic target [59]. RUNX1 is highly expressed in lung cancer and correlates with poor prognosis [60]. Studies suggest RUNX1 promotes migration and proliferation, indicating its potential tumor-suppressive function [57]. In NSCLC, RUNX1 plays a crucial role in the macrophage–myofibroblast transition (MMT) pathway. Increased levels of RUNX1 positively correlate with the abundance of cancer-associated fibroblasts (CAFs) derived from MMT and with patient mortality [61]. Targeting TGFB1/SMAD3/RUNX1 signaling inhibits MMT-driven CAF and tumor formation in NSCLC. A study showed that silencing RUNX1 inhibits lung cancer progression via the ERK/MAPK axis by modulating ACP5 expression [62]. These results suggest RUNX1 as a potential therapeutic target.

Peroxisome proliferator-activated receptor gamma (PPARG), a ligand-activated nuclear transcription factor, plays a pivotal role in regulating gene expression and is known for its anti-inflammatory and anti-proliferative effects in various cancers [63,64]. Studies have demonstrated that PPARG is a tumor suppressor in NSCLC [65]. Decreased PPARG expression in human lung tumors correlates with poor prognosis, while increased expression influences the production of cytokines crucial for angiogenesis and tumor–stromal interactions [66]. PPARG exhibits a multifaceted effect in lung cancer by influencing various molecular mechanisms and impacting the entire tumor microenvironment (TME), including noncancerous elements such as immune cells, fibroblasts, adipocytes, and vascular components [67]. PPARG ligands induce differentiation and apoptosis in NSCLC cells [68]. Its activation inhibits angiogenesis, suppresses fibroblast differentiation into myofibroblasts, and remodels the extracellular matrix, creating a less supportive TME [69,70,71]. PPARG activation suppresses cancer cell spread by inhibiting epithelial–mesenchymal transition (EMT) and blocking TGFB-induced EMT, ultimately reducing the metastatic potential of cancer cells [72]. These findings underscore the therapeutic potential of targeting PPARG in lung cancer. Moreover, targeting PPARG-related metabolic adaptations in hypoxic tumor cells holds potential for overcoming chemoresistance and improving outcomes in NSCLC [73].

The transforming growth factor beta (TGFB) is a multi-functional cytokine and has dual effects on cancer progression, including NSCLC [74]. TGFB signaling regulates cell growth, differentiation, and death in a wide range of biological processes, including tumor initiation and progression [75]. TGFB plays a crucial role in cancer metastasis by activating EMT, while it also regulates the cell cycle by inducing the expression of CDK inhibitor p21 and causing G1 arrest [76]. TGFB ligands initiate signals by binding to the TGFB type II receptor (TGFBR2), which is known to play a crucial role in tumor progression by promoting tumor growth, invasion, and metastasis, while suppressing immune responses [77,78]. The inhibition of TGFBR2 has been shown to induce an immune response and promote tumor regression in certain cancers [79]. This suggests that targeting TGFBR2 or its downstream signaling pathways may represent a potential therapeutic strategy.

The analysis of CNVs offers a spectrum of possibilities related to pathogenesis, the monitoring of progression, treatment, and prognosis in lung cancer. This research identified genetic abnormalities, followed by a functional analysis of individual genes, which provided a more detailed picture of lung cancer etiology. Although the small number of patients is a limitation of the study, strict selection criteria allowed the selection of a cancerous and homogenous group, making the study noteworthy, especially when the results aim to serve as preliminary data for further studies. In conclusion, this study enhances our understanding of the molecular mechanisms of lung cancer. Leveraging gene expression profiling techniques like oligonucleotide microarrays enables precise classification. Our findings highlight large genomic abnormalities in NSCLC samples, including CNVs involving common regions like 3p, 5q, 13q, and 21q, along with gain in the 12p region. Our analysis of oncogenes identified 59 commonly absent genes in these regions, with GO enrichment analysis pointing to several terms closely associated with lung cancer. These results shed light on the genomic landscape of NSCLC and its implications for disease pathogenesis, encouraging us to expand the research to a larger study group.

## 4. Materials and Methods

The study group included 60 patients—49 men and 11 women. All of them gave their written consent for participation in the study. From this group of patients, three representatives were selected based on histochemical staining. To obtain the most authoritative results, only patients with an amount of cancer cells equal or higher than 80% were subjected to genetic analysis, creating a highly cancerous, homogenous population.

### 4.1. Histochemical Staining with Hematoxylin and Eosin (H + E)

We performed histological hematoxylin and eosin staining to confirm the presence of tumor infiltration in the lung tissue and determine the tumor-to-stroma ratio. Tissue sections were cut from frozen tumor samples into glass slides using a cryostat. These sections were dried under a fan for 10–15 min. Subsequently, the slides were stained with hematoxylin (Merck, Darmstadt, Germany) for 7 min, rinsed under running tap water for 10 min, stained with eosin (Merck, Darmstadt, Germany) for 2 min, and then rinsed with water. The slides underwent a series of alcohol dehydrations—50%, 70%, and 90%, corresponding to absolute alcohol, alcohol + xylene, and xylene. The preparations were then mounted with Canada balsam (Merck, Darmstadt, Germany) under a coverslip. The cancer cell content in individual tissues was assessed under a light microscope (Leica, Wetzlar, Germany). Only tissues with a content of cancer cells of 80–90% were selected for further analysis.

### 4.2. Patient Characterization 

#### 4.2.1. Patient No. 1

A 75-year-old man underwent a thoracotomy, during which a 52 mm tumor was excised. The histopathological examination revealed planoepithelial cancer (G2, T2b, N2, M0), corresponding to stage 3a according to the TNM classification. There were complications in the post-operative period. The patient was discharged on the 36th day after the surgery.

Medical history: the patient smoked for 54 years, about 30 cigarettes per day. At the time of the operation, the patient had stopped smoking for three years. There was no family history of cancer.

Comorbidities: chronic obstructive pulmonary disease, coronary artery disease in hypertension, coronary heart disease, atrial fibrillation, and hyperthyroidism.

#### 4.2.2. Patient No. 2

A 68-year-old man underwent thoracotomy, during which a tumor of 49 mm in size was removed. The histopathological examination revealed planoepithelial cancer (G2, T2a, N0, and M0), corresponding to stage 1b according to the TNM classification. There were no complications in the postoperative period. After six days of inpatient treatment, the patient was discharged in good general condition.

Medical history: despite the detection of the tumor, the patient remained an active smoker; he has smoked for 36 years, about 27 cigarettes per day. There was no family history of cancer.

Comorbidities: none/not reported.

#### 4.2.3. Patient No. 3

A 58-year-old man underwent thoracotomy, during which a tumor with a diameter of 38 mm was removed. The histopathological examination revealed planoepithelial cancer (G2, T2a, N1, and M0), corresponding to stage 2a according to the TNM classification. There were no complications in the post-operative period. The patient was discharged from the hospital on the 5th day after surgery in good general condition.

Medical history: the patient smoked for 35 years, about 40 cigarettes per day. There was no family history of cancer.

Comorbidities: hypertension.

### 4.3. DNA Microarray Analysis 

Once the treatment was completed, we performed advanced molecular testing to identify tumor-specific genomic changes. Briefly, the Genome-Wide Human CytoScan HD Array and CytoScan 750K (Affymetrix, Santa Clara, CA, USA) were used to analyze the genomic alterations in the tumor sample. After initial homogenization and proteinase K treatment (final conc. 1 mg/mL, 55 °C, 2 h), genomic DNA was obtained from frozen sections using the column method (DNeasy Blood & Tissue Kit, Qiagen, Hilden, Germany). Next, 250 ng of the genomic DNA from the tumor tissue was subjected to microarray examination according to the manufacturer’s protocols.

All used consumables were provided by the manufacturer. Concentrations of enzymes were not given. Initially, the DNA was digested using the NspI restriction enzyme (37 °C for 2 h, followed by 65 °C for 20 min) and subsequently ligated to an adapter (16 °C for 3 h, 70 °C for 20 min). A PCR amplification was performed using a single pair of primers targeting the adapter sequence and Titanium™ Taq DNA Polymerase (Takara, Japan) as follows: initial denaturation at −94 °C for 3 min (1×, cycling at −94 °C for 30 s, annealing 60 °C for 45 s (30×), and a final elongation at 68 °C for 15 s. The resulting PCR products were then analyzed on a 2% TAE gel to ensure that most products fell within the 150 to 2000 bp range (1 Kb marker, Invitrogen, Waltham, MA, USA). PCR products from each sample (4 independent reactions) were combined and purified using magnetic beads at 1.5× volume ratio (Agencourt AMPure, Beckman Coulter, Beverly, MA, USA). The purified PCR products were fragmented using a Fragmentation Reagent (Affymetrix, Santa Clara, CA, USA) as follows: 45 μL of purified PCR product was combined with 10 μL Fragmentation Master Mix and incubated at 37 °C for 35 min. Fragmentation reaction was stopped by incubation at 95 °C for 15 min. DNA was visualized on a 4% TAE agarose gel to confirm that the fragment sizes ranged from 25 to 150 bp (50 bp marker, Invitrogen, Waltham, MA, USA). The fragmented PCR products were subsequently end-labeled with biotin (2 μL DNA Labeling Reagent and 3.5 μL TdT Enzyme incubated at 37 °C for 4 h and 95 °C for 15 min) and hybridized to the array at 50 °C, 60 rpm for 16 to 18 h. The arrays were then washed and stained using a GeneChip^®^ Fluidics Station 450 (Thermo Fisher Scientific, Waltham, MA, USA) (implemented protocol) and scanned using an Affymetrix GeneChip^®^ Scanner 3000 7G. The scanned data files were generated using Affymetrix GeneChip Command Console Software, version 1.2, and analyzed with Affymetrix^®^ Chromosome Analysis Suite v 2.0.0.195 (ChAS) (Affymetrix, Santa Clara, CA, USA).

To calculate the copy number variations (CNVs), the data were normalized to baseline reference intensities using the Affymetrix model NA 32.3. The Hidden Markov Model (HMM) available within the software package was used to determine the copy number states and their breakpoints. Thresholds of log2ratio ≥ 0.58 and ≤−1 were used to categorize altered regions as CNV gains (amplification) and copy number losses (deletions), respectively. To prevent the detection of false-positive CNVs arising due to inherent microarray “noise”, only alterations that involved at least 25 consecutive probes and that were bigger than 50 Kbp in length were considered in the analysis of gains or losses in our study. Amplifications and deletions were analyzed separately. To exclude aberrations representing common CNVs, all of the identified CNVs were compared with those reported in the Database of Genomic Variants (DGV, https://ngdc.cncb.ac.cn/databasecommons/database/id/283 (accessed on 2 August 2024)). To further identify the genes involved in the CNVs, we used the UCSC database (http://genome.ucsc.edu (accessed on 3 August 2024)) and Ensembl (http://www.ensembl.org (accessed on 3 August 2024)). Gene annotation and gene overlap were determined using the human genome build 19 (hg19) and NetAffx (http://www.affymetrix.com (accessed on 3 August 2024)). For LOH analysis, we utilized the algorithm incorporated in the ChAS software v4.5. As matched normal DNA was available for all of our samples, a matched-pair analysis was employed to detect LOH and cnLOH (copy neutral LOH, without a change in the copy number). Regions of LOH/cnLOH that were >3 Mbp were identified. The obtained results derived from both algorithms were fully concordant. The acquired data were finally checked for the presence in the Integrated Genomic Database of Non-Small Cell Lung Carcinoma (https://ngdc.cncb.ac.cn/databasecommons/database/id/547 (accessed on 3 August 2024)).

### 4.4. Identification of LOH Lesions 

To further elucidate the causativeness of identified abnormalities and emerge pathways governing neoplasm in studied patients, we searched the regions for genes with established roles in cancer development. The COSMIC v95 database (https://cancer.sanger.ac.uk/ (accessed on 3 August 2024)) and Cancer Gene Census catalogue were used, and the gene list was retrieved as a BED file. This file was then imported to Affymetrix^®^ ChAS software v4.5, and genes encompassed by regions of interest were extracted.

### 4.5. Functional Analysis

Genes form Cancer Gene Census were functionally analyzed using the Metascape bioinformatic tool [80,81]. The human Entrez Gene IDs for these genes were retrieved using the “biomaRt” package in the R programming language [82]. These IDs were subsequently analyzed in Metascape to identify statistically enriched terms. The analysis included calculating accumulative hypergeometric *p*-values and enrichment factors, which were used to filter the results. Significant enriched terms were hierarchically clustered based on kappa-statistical similarities among the associated genes, using a kappa score threshold of 0.3 to define term clusters. The top 20 enriched term clusters from all statistically significant ontology terms were visualized in a bar graph. Additionally, the genes in the three most significantly enriched clusters were visualized using Cytoscape (v. 3.7.2) [83]. Metascape was also employed to search the TRRUST database (Transcriptional Regulatory Relationships Unravelled by Sentence-based Text mining, version 2). The interaction between transcription factors and genes was shown using Cytoscape.

## Figures and Tables

**Table 1:** Identified genomic rearrangements in patient no. 1.

Type	CN	Chromosome	Locus	Start (nt)	End (nt)	Size (kb)	Gene Count, Total
Gain	2.32	2	p25.3	12,770	71,741,865	71,729.095	434
Gain	2.22	8	q23.1	107,766,798	127,017,270	19,250.472	73
Gain	2.33	9	p22.3	16,129,629	80,872,801	64,743.172	277
Gain	2.46	9	p24.3	208,454	3,571,087	3362.633	12
Gain	2.34	11	q13.2	67,021,050	108,714,926	41,693.876	317
Gain	2.68	12	p13.33	173,786	11,813,033	11,639.247	203
Gain	2.41	14	q32.2	96,975,842	107,284,437	10,308.595	188
Gain	2.85	15	q26.1	91,665,391	102,429,040	10,763.649	51
Gain	2.53	X	q22.3	107,900,776	155,233,098	47,332.322	386
Gain	1.04	Y	p11.31	2,650,424	28,799,654	26,149.23	102
Loss	1.64	1	p21.3	99,380,695	150,966,503	51,585.808	304
Loss	1.50	3	p26.3	61,891	94,775,034	94,713.143	599
Loss	1.49	4	q34.1	175,499,137	190,957,460	15,458.323	66
Loss	1.50	5	q11.2	56,978,186	180,715,096	123,736.91	807
Loss	1.66	6	q11.1	62,094,327	95,241,423	33,147.096	109
Loss	1.68	7	p14.1	37,590,599	70,784,719	33,194.12	168
Loss	1.67	8	q24.22	133,534,264	146,295,771	12,761.507	132
Loss	1.56	9	q21.11	71,249,423	98,423,588	27,174.165	159
Loss	1.50	10	q21.1	53,123,032	95,409,107	42,286.075	258
Loss	1.53	13	q11	19,436,286	115,107,733	95,671.447	459
Loss	1.48	14	q12	31,988,029	92,706,803	60,718.774	369
Loss	1.34	18	q21.33	59,806,229	78,013,728	18,207.499	72
Loss	1.53	21	q11.2	15,016,486	48,093,361	33,076.875	292

**Table 2:** Identified genomic rearrangements in patient no. 2.

Type	CN	Chromosome	Locus	Start (nt)	End (nt)	Size (kbp)	Gene Count, Total
Gain	2.33	1	p13.1	117,476,258	165,134,313	47,658.055	492
Gain	2.56	1	q41	218,274,740	249,224,684	30,949.944	262
Gain	2.89	2	p25.3	12,770	102,380,127	102,367.357	648
Gain	2.22	2	q11.2	100,346,874	120,068,585	19,721.711	131
Gain	2.36	6	p12.1	54,557,513	99,206,915	44,649.402	134
Gain	2.51	6	q25.2	154,755,476	170,914,297	16,158.821	100
Gain	2.22	7	q21.11	80,254,491	96,327,224	16,072.733	74
Gain	2.24	7	q34	140,961,678	159,119,707	18,158.029	180
Gain	2.73	9	p13.2	36,366,584	141,018,648	104,652.064	749
Gain	2.25	10	p14	10,274,286	37,497,811	27,223.525	144
Gain	2.34	10	q25.3	118,838,312	135,426,386	16,588.074	133
Gain	2.68	11	p14.3	22,621,011	45,987,869	23,366.858	102
Gain	2.66	12	p13.33	173,786	133,777,562	133,603.776	1184
Gain	2.45	16	q21	60,600,185	80,068,006	19,467.821	181
Gain	2.29	17	q12	32,417,015	54,939,625	22,522.61	465
Gain	2.67	18	p11.32	136,227	8,877,061	8740.834	46
Gain	2.57	19	p13.3	260,911	58,956,816	58,695.905	1621
Gain	2.61	22	q11.21	19,709,385	41,851,930	22,142.545	358
Gain	2.21	22	q12.3	35,493,366	50,899,310	15,405.944	246
Loss	1.53	1	p36.33	854,277	120,118,291	119,264.014	1215
Loss	1.60	2	q22.1	141,698,982	242,782,258	101,083.276	629
Loss	1.56	3	p26.3	61,891	84,844,800	84,782.909	580
Loss	1.51	4	p16.3	68,345	164,845,295	164,776.95	786
Loss	1.54	5	q11.2	50,942,623	180,715,096	129,772.473	844
Loss	1.60	6	p25.3	302,260	56,509,337	56,207.077	701
Loss	1.73	7	p22.3	43,376	7,872,835	7829.459	91
Loss	1.65	9	p24.3	208,454	25,954,669	25,746.215	107
Loss	1.19	10	q21.1	53,733,770	73,299,253	19,565.483	85
Loss	1.68	11	p15.5	230,680	13,741,855	13,511.175	283
Loss	1.63	11	q11	55,590,295	97,097,033	41,506.738	622
Loss	1.21	13	q13.1	32,239,432	115,107,733	82,868.301	362
Loss	1.27	15	q11.2	22,770,421	78,426,298	55,655.877	606
Loss	1.75	15	q25.1	79,047,350	92,596,186	13,548.836	140
Loss	1.58	16	p13.3	6,123,936	52,234,735	46,110.799	347
Loss	1.57	17	q21.33	49,307,721	81,041,823	31,734.102	410
Loss	1.57	18	q11.2	19,471,272	72,422,615	52,951.343	220
Loss	1.65	20	q11.23	34,459,007	57,404,888	22,945.881	230
Loss	1.67	21	q11.2	15,016,486	44,847,203	29,830.717	220
Loss	1.26	X	p22.33	168,551	155,233,098	155,064.547	1020

**Table 3:** Identified genomic rearrangements in patient no. 3.

Type	CN	Chromosome	Cytoband Start	Start (nt)	End (nt)	Size (kbp)	Gene Count, Total
Gain	2.3453367	1	p36.21	14,927,444	112,538,743	97,611.299	936
Gain	2.8998601	2	q11.2	100,146,735	220,003,241	119,856.506	648
Gain	2.8915234	6	p25.3	156,974	75,934,361	75,777.387	744
Gain	2.419764	6	q14.1	80,753,066	170,914,297	90,161.231	458
Gain	2.2164984	7	q35	146,158,986	159,119,707	12,960.721	111
Gain	2.6335676	7	q36.2	154,894,170	159,119,707	4225.537	26
Gain	2.5188828	8	q21.13	80,890,358	146,295,771	65,405.413	368
Gain	2.904787	9	p24.3	208,454	36,614,229	36,405.775	214
Gain	2.369618	12	p13.33	173,786	29,825,544	29,651.758	313
Gain	2.2530286	12	q13.3	57,858,615	88,360,183	30,501.568	142
Gain	2.3361242	12	q21.33	90,629,021	133,777,562	43,148.541	382
Gain	2.5275028	15	q24.3	78,130,041	102,429,040	24,298.999	207
Gain	2.319723	16	p13.3	85,880	18,990,301	18,904.421	298
Gain	2.3445287	17	p13.3	525	49,233,446	49,232.921	964
Gain	2.4188483	18	p11.32	136,227	78,013,728	77,877.501	342
Gain	2.455756	20	q11.23	36,467,743	62,913,645	26,445.902	310
Loss	1.6451818	2	p25.2	5,860,401	82,122,733	76,262.332	480
Loss	1.7397279	2	q36.3	228,226,071	242,782,258	14,556.187	161
Loss	1.6913146	3	p26.3	61,891	64,958,024	64,896.133	538
Loss	1.6292121	4	p15.33	11,628,372	190,957,460	179,329.088	745
Loss	1.640046	5	q12.3	65,095,664	180,715,096	115,619.432	778
Loss	1.6604121	7	p22.3	43,376	149,397,765	149,354.389	1050
Loss	1.1793071	8	p23.3	158,048	64,152,758	63,994.71	387
Loss	1.6649909	9	q21.13	74,208,998	130,535,660	56,326.662	403
Loss	1.6489277	10	p15.3	100,047	64,303,578	64,203.531	343
Loss	1.6821278	10	q23.1	84,735,681	119,789,550	35,053.869	313
Loss	1.6813701	11	p15.5	230,750	123,097,931	122,867.181	1355
Loss	1.6759429	12	p11.21	31,356,130	55,346,490	23,990.36	243
Loss	1.55947	13	q11	19,436,286	114,003,154	94,566.868	443
Loss	1.6753877	15	q11.2	22,770,421	69,986,486	47,216.065	502
Loss	1.7330544	16	p12.2	23,237,480	54,395,215	31,157.735	222
Loss	1.7394392	16	q12.2	55,169,214	81,505,954	26,336.74	269
Loss	1.7052059	19	p13.3	260,911	58,956,816	58,695.905	1621
Loss	1.6436315	21	q11.2	15,016,486	40,428,341	25,411.855	169
Loss	1.6629032	22	q11.1	16,888,899	51,197,766	34,308.867	544

## Data Availability

The raw data supporting the conclusions of this article can be made available by the authors on request.
